# Incidence of blindness in open-angle glaucoma in Sweden: a long-term follow-up study

**DOI:** 10.48101/ujms.v129.10664

**Published:** 2024-10-28

**Authors:** Curt Ekström, Christoffer Carlsson

**Affiliations:** Department of Surgical Sciences, Ophthalmology, Uppsala University, Uppsala, Sweden

**Keywords:** Blindness, cohort study, epidemiology, health care, male sex, open-angle glaucoma, pseudoexfoliation, risk factor

## Abstract

**Background:**

Open-angle glaucoma (OAG) is a leading cause of irreversible blindness. There are no prospective studies on the risk of developing blindness in both eyes in individuals with definite OAG.

**Methods:**

A total of 354 patients with newly diagnosed OAG, who had participated in four studies conducted at the Eye Department in Tierp, Sweden, from 1979 to 2006, were included in the investigation. Using the World Health Organization’s criteria for blindness, medical records, glaucoma case records, and visual fields were reviewed to identify patients who developed bilateral blindness. Incidence proportions and incidence rates were estimated. To assess potential risk factors for blindness, standardised morbidity ratios (SMRs) were calculated. The effects of age and sex were also analysed using Cox proportional hazard models.

**Results:**

By the end of the study in August 2023, 33 cases of blindness caused by OAG had been found, corresponding to an incidence proportion of 9.3% (95% confidence interval [CI]: 6.5–12.8%). Within the first 20 years, 29 cases were detected, yielding a proportion of 8.2% (95% CI: 5.5–11.6%). The incidence rate was estimated to be 8.6 per 1,000 person-years (95% CI: 5.9–12.6 per 1,000 person-years). Glaucoma-related blindness was associated with male sex (SMR 2.33; 95% CI: 1.13–4.80). The hazard ratio was doubled for every 5 year of increasing age (2.21; 95% CI: 1.60–3.05).

**Conclusion:**

In this study of blindness in newly diagnosed OAG in a Swedish population, approximately one in 10 patients progressed to bilateral blindness caused by the disease. Old age and male sex were identified as significant risk factors.

## Introduction

Open-angle glaucoma (OAG) is an age-related neurodegenerative disease of significant importance for public health, characterised by the progressive loss of optic nerve fibres, resulting in cupping of the optic disc and consistent visual field defects. Although glaucoma is a leading cause of irreversible blindness worldwide (1), only 15% or fewer of those with OAG are estimated to progress to end stage disease (2). In African and European-derived populations, OAG is the predominant form of glaucoma, while angle-closure glaucoma is commonly found in Asian populations. The latter form of glaucoma is believed to have a worse prognosis than OAG (3). In Sweden, increased intraocular pressure (IOP) and pseudoexfoliation (PEX) are important risk factors for the development of OAG (4).

Several studies, mainly conducted on European-derived populations, have reported the incidence of blindness in OAG, with conflicting results. Five of these are shown in Table 1 (5–9). Some studies were based on data register searches (5, 9), or review of medical records (6), while others used data from glaucoma patients who had died while under follow-up in clinical practice (7, 8, 10, 11). In four out of the five studies in Table 1, Kaplan-Meier survival curves were used to calculate the cumulative probability of blindness, one of which was adjusted for competing events.

**Table 1:** Incidence of bilateral blindness in open-angle glaucoma across five studies.

Study	Reference	No. of Patients	Incidence	Time Years
Olmsted, Minnesota	(5)	100	22% ^a^	20
Seattle, Washington	(6)	186	6% ^a^	15
Ekenäs, Finland	(7)	106	6% ^b^	20
Malmö, Sweden	(8)	592	13.5% ^c^	20
Olmsted, Minnesota	(9)	563	4.3% ^a^	20

a Kaplan-Meier cumulative probability.

b Incidence proportion.

c Kaplan-Meier cumulative probability, adjusted for competing events.

The World Health Organizations criteria for blindness was used in the Swedish study; the other studies used the United States’ criteria for legal blindness.

The intention of the present research was to estimate the risk of blindness in subjects with definite OAG in two rural districts in Sweden. For this purpose, individuals diagnosed with OAG in four glaucoma investigations were included in a long-term follow-up study.

## Methods

### Eligibility

The eligibility criteria for entry into the study included being a resident in one of the two rural districts of Tierp or Älvkarleby in the north of Uppsala County, south central Sweden, and being diagnosed with OAG in studies undertaken at the Eye Department, Tierp between 1979 and 2006. These studies consisted of: 1) 224 patients identified in a study on new cases of OAG in the north of Uppsala County (12, preliminary results); 2) 22 individuals with untreated OAG who participated in a population survey in Tierp (13); 3) 46 individuals detected in the follow-up of the population survey (4); and 4) 134 patients diagnosed in a case-control study on incident OAG in clinical practice (14).

### The study cohort

**Figure 1:**
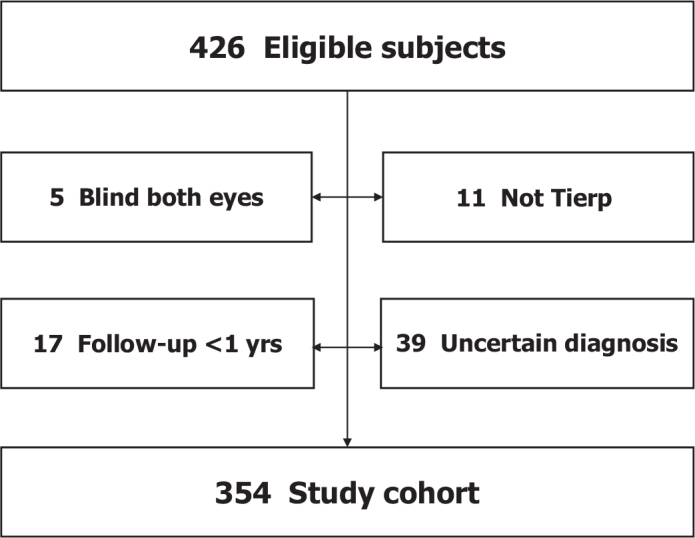
Flowchart showing how the study cohort of 354 individuals was derived. Not Tierp = not examined in Tierp.

Out of the 426 individuals eligible for the study, five were blind on both eyes at presentation and subsequently excluded. Eleven patients were not examined in Tierp, and 17 had a follow-up time of less than 1 year. These patients were excluded, as were 39 individuals who did not meet the criteria for a definite diagnosis of OAG (Figure 1). The remaining 354 people constituted the study cohort, whose characteristics are shown in Table 2. The study was approved by the Regional Ethical Review Board of Uppsala University (Dnr 2012/428/1), and the principles of the Declaration of Helsinki were observed.

**Table 2:** Characteristics of the cohort, by age at diagnosis and sex.

Age	No. (*n* = 354)		Person years (*n* = 3,845)	
	Females (%)	Males (%)	Females (%)	Males (%)
45–64 years	23 (12)	17 (11)	481 (22)	377 (23)
65–74 years	73 (37)	74 (47)	906 (41)	878 (53)
75–84 years	89 (45)	62 (39)	749 (34)	382 (23)
≥ 85 years	11 (6)	5 (3)	58 (3)	15 (1)
Total	196 (100)	158 (100)	2,194 (100)	1,652 (100)

Note: Mean age = 74.1 years (standard deviation = 7.1 years).

### Classification of open-angle glaucoma

Consistent with the concept proposed by Foster et al. (15), glaucoma accompanied by PEX was classified as OAG. The occurrence of PEX was recorded if observed at the time of diagnosis or within 2 years thereafter. To qualify for a diagnosis of OAG, a repeatable visual field defect in either eye was a prerequisite, consistent with glaucoma and not attributable to other causes. Visual field tests were conducted using the Competer 350 automated perimeter (Bara Elektronik AB, Lund, Sweden), and the Haag-Streit Goldmann perimeter, as described in details elsewhere (13, 16). Nineteen subjects either had only one visual field test or did not fully comply with the testing. Nevertheless, they were counted as having OAG based on the following criteria: 1) repeated IOP readings ≥ 30 mmHg and an optic disc excavated to the disc margin (*n* = 7); 2) repeated IOP readings ≥ 35 and a glaucomatous disc that was not excavated to the margin (*n* = 10); and 3) IOP ≥ 40 and a dense cataract (*n* = 2).

### Registration of blindness

Glaucoma patients were followed with repeated eye examinations, which included registering best-corrected visual acuity and conducting visual field tests. To assess incident cases of blindness, glaucoma case records, medical records, and visual fields from the Tierp Health Centre and Uppsala University Hospital were reviewed. The criteria set by the World Health Organization (WHO) for blindness were used (17). Thus, blindness was defined as having a visual acuity < 0.05, or a visual field no wider than 10° around central fixation, using the Goldmann III4e test object or a stronger stimulus, in the better eye. Almost all individuals who fulfilled the visual field criteria were examined with the Goldmann perimeter.

### Assessment of systemic predictors

Information regarding treated systemic hypertension and ischaemic heart disease was obtained during interviews or retrieved from medical records. In cases where there was a discrepancy between the self-reported history and the medical record, data from the latter source were used in this report. Participants were asked whether they were current smokers or past smokers and when they stopped smoking. Information was also acquired from medical records and family members.

### Statistical methods

Using the binomial and Poisson distributions, respectively, incidence proportions and rates, together with their 95% confidence intervals (CIs), were estimated. Stratum specific incidence rates and their difference were also calculated. Follow-up time was determined from the date of OAG diagnosis to the date of blindness in both eyes (*n* = 60), date of non-compliance with eye examinations (*n* = 12), date of losses to follow-up (*n* = 275), date of migration out of Uppsala County (*n* = 6), or the end of the study (*n* = 1), whichever occurred first. Incidence rates were converted to risks using 5-year intervals (18).

Age-standardised morbidity ratios (SMRs) were computed to evaluate potential risk factors for blindness. Subsequently, Cox proportional hazards models were developed to further assess the effects of age and sex, with adjustment for competing events. The proportionality assumption was tested using time-dependant variables, indicating that the effect of covariates on survival was independent of time. Finally, Kaplan-Meier surviving curves were created to illustrate the difference between females and males.

## Results

At baseline, PEX was present in 220 patients (62.1%). The median follow-up time was 9.3 years (range 1–35 years). By the end of the study on 31 August 2023, 33 cases of bilateral blindness with OAG as the main cause had been found, resulting in an incidence proportion of 9.3% (95% CI: 6.5–12.8%). Within the first 20 years, 29 cases were detected, yielding a proportion of 8.2% (95% CI: 5.5–11.6%). The incidence rate was estimated to be 8.6 per 1,000 person-years (95% CI: 5.9–12.6 per 1,000). Standardisation to the mean population in Tierp in 1994 produced identical results. The rate was higher in people aged ≥ 75 years and in males (Table 3). The incidence rate for the first 20 years was 8.0 per 1,000 person-years, which may be converted to an approximate risk of 9%. Blindness in both eyes was detected in 60 individuals, with OAG being the most common cause, followed by macular diseases (*n* = 11), and cataract (*n* = 5). Nine individuals had different causes in their two eyes, all of whom had OAG as the main cause in one eye.

**Table 3:** Incidence rate of blindness in open-angle glaucoma per 1,000 person-years at risk, by age at diagnosis and sex.

Age	Females		Males		All	
	Rate	(95% CI)	Rate	(95% CI)	Rate	(95% CI)
< 75 years	5.0	(2.0–10.4)	8.0	(3.8–14.7)	6.4	(3.7–10.3)
≥ 75 years	6.2	(2.0–14.5)	27.7	(13.8–49.6)	13.3	(7.6–21.6)
Total	5.5	(2.8–9.6)	12.7	(7.9–19.4)	8.6	(5.9–12.1)

Note: Difference males – females = 8.7 (95% CI – 2.2 – 19.6), standardised to the distribution of person-years in all participants.

CI: Confidence interval.

**Figure 2:**
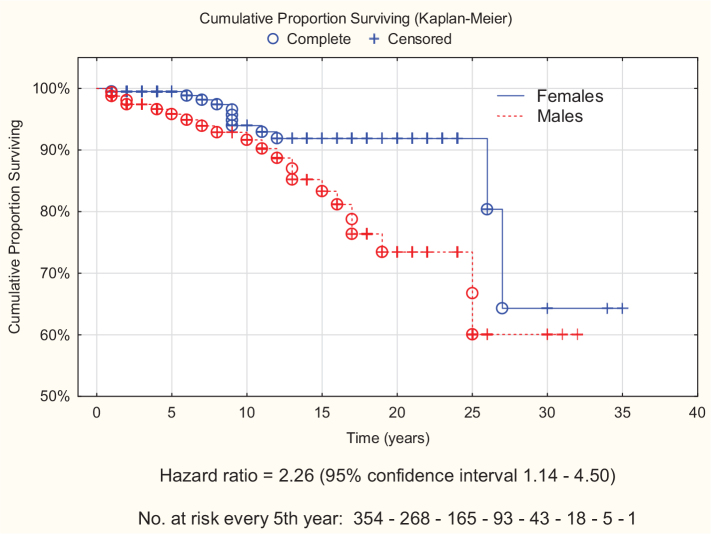
Kaplan-Meier survival curves for bilateral blindness in 354 individuals with open-angle glaucoma, by sex.

As shown in Table 4, male sex increased the risk of OAG-related blindness, while advanced age, PEX, smoking status, systemic hypertension, and ischaemic heart disease did not show significant associations. A Cox regression model including age and sex revealed a 3-fold increased risk in men (hazard ratio [HR] 2.95; 95% CI: 1.43–6.06), while every 5-year increase in age increased the risk more than 2-fold (HR 2.21; 95% CI: 1.60–3.05). Adding PEX into the model yielded almost identical results, with a HR for PEX of 0.70 (95% CI: 0.35–1.40). There was no sign of interaction between age and sex. Adjustment for competing events (losses to follow-up) had no effect on the estimates. Survival curves illustrating the relationship between sex and OAG-related blindness are presented in Figure 2.

**Table 4:** Potential risk factors for blindness in open-angle glaucoma, standardised for age.

Characteristics		No. of cases (*n* = 33)	SMR	(95% CI)
Age ≥ 75 years ^a^	No	17	1.00	
	Yes	16	1.95	(0.97–3.90)
Male sex	No	12	1.00	
	Yes	21	2.33	(1.13–4.80)
PEX, either eye	No	16	1.0	
	Yes	17	0.66	(0.33–1.31)
Smoking status	Never smoked	23	1.00	
	Past smoker	5	0.76	(0.29–1.99)
	Current smoker	5	1.75	(0.66–4.67)
Hypertension, treated	No	25	1.00	
	Yes	8	0.61	(0.27–1.35)
Ischaemic heart disease	No	23	1.00	
	Yes	10	2.05	(0.96–4.37)

a Age at diagnosis, standardised for sex.

CI: confidence interval; PEX: pseudoexfoliation; SMR: standardised morbidity ratio.

## Discussion

The main finding of this long-term follow-up study on OAG, covering up to 35 years, in two rural districts in Sweden, was the relatively low incidence of blindness. In the first 20 years, the incidence rate was 8.0 per 1,000 person-years, roughly corresponding to a risk of 9%. Our cohort was composed of individuals newly diagnosed with definite OAG across four separate studies. Thus, the characteristic of the present study differed from other studies, based on the search of data registers (5, 9), or data from patients who had passed away during follow-up in clinical practice (7, 8).

Direct comparisons with previous studies of OAG blindness pose several challenges. Firstly, many studies have included patients with ocular hypertension (7, 9). Patients with treated ocular hypertension are typically better off than those with manifest glaucoma, which give rise to selection bias. Secondly, some studies have had small sample sizes (6, 7), contributing to uncertainty in the estimates. Thirdly, Kaplan-Meier survival analysis has frequently been used to calculate estimates for blindness (5, 6, 9), although it is well known that this may overestimate the results (19). Finally, there is no standardised definition for blindness, which may explain some of the variation in the results (20). We have not found any study reporting the incidence rate, which is the method of choice when comparing different studies.

In the present investigation, the incidence proportion at the conclusion of the study was 9.3%. Death was the endpoint for 218 individuals, followed by non-attendance in 57 individuals, a sizeable majority of whom died shortly after missing their appointments. Clearly, individuals who died could not develop blindness from glaucoma. Only 12 individuals failed to comply with visual acuity testing, and six emigrated. Therefore, we believe that the incidence proportion of nearly 10% is a good estimate of the risk of blindness in individuals with OAG. As mentioned above, making comparisons with other studies is challenging. Nevertheless, a study conducted in Malmö (8), southern Sweden, reported a cumulative incidence of 13.5%, adjusted for competing events, after 20 years, a result quite similar to that observed in Tierp. Both studies used the WHO criteria of blindness.

The association between older age and blindness, as reported in other studies (5, 9), was confirmed in the present study. However, contrary to previous research (5, 8, 9), men had a 3-fold increased rate of blindness, compared to women. This finding was consistent across both the stratified as well as the multivariable analyses, with or without adjustment for competing events. Bearing the strong association in mind, it is unlikely that unknown confounders can explain this result. Pseudoexfoliation has been found to increase the risk of blindness in some studies (7), but not in others (21). In the present study, PEX did not increase the risk.

Our study has several strengths, including its community-based design, sizeable cohort, and long-term follow-up. The cohort comprised individuals with OAG, detected in four investigations undertaken at the Eye Department in Tierp, and they were followed with the aim of examining the prognosis of OAG. All baseline eye examinations were conducted by the same glaucoma specialist. Furthermore, for an OAG diagnosis, a repeatable visual field defect or end-stage disease in either eye was required. Other criteria were used for the inclusion of 19 patients, as described in the Introduction section. However, it is most likely that all of these individuals had definite OAG. Nonetheless, as with many epidemiologic studies, our research was limited in some respects.

Most importantly, even if the cohort comprised more than 3,800 person-years at risk, the number of cases were not more than 33, which limited the power to provide reliable estimates on some of the possible predictors for blindness. Thus, a relationship between diabetes and glaucoma has been reported (22), but none of the cases in our study were diagnosed with diabetes. Furthermore, individuals diagnosed with ischaemic heart disease had a doubled risk of blindness, although the CI was wide. Secondly, there is a risk of misclassification of exposure when data are based on medical records or self-reports, as was the case with smoking habits. However, it is worth noting that the information on exposure was collected before the outcome of the study. Consequently, this type of bias should be non-differential, thereby ‘diluting’ the relationship between glaucoma blindness and possible predictors. It is possible that some cases may have been missed. Nevertheless, by applying repeated testing of visual fields and visual acuity, we believe that misclassification of disease was a minor problem in this study.

A total of 54 individuals who participated in the population survey in Tierp were included in the cohort. It is well known that glaucoma patients identified in screening studies tend to have better visual fields than those detected in clinical practice (23). Similarly, results have suggested that the former category may have a better prognosis (24). In fact, in our study, patients diagnosed in clinical practice had a 2-fold increased risk of blindness. Clearly, the results of these types of studies are dependent on the characteristics of those involved. With respect to the low number of individuals, excluding participants from the population survey would only marginally affect the estimates.

While therapeutic advancements in OAG are obvious in recent decades, a smaller proportion of patients may still progress to blindness in both eyes. In this investigation on blindness in newly diagnosed OAG in two rural districts in Sweden, approximately one out of 10 patients was blind at the end of the study. Old age and male sex increased the risk.
